# Human umbilical cord stem cell conditioned medium versus serum-free culture medium in the treatment of cryopreserved human ovarian tissues in in-vitro culture: a randomized controlled trial

**DOI:** 10.1186/s13287-017-0604-4

**Published:** 2017-06-24

**Authors:** Yingxian Jia, Xiaohan Shi, Yidong Xie, Xiaochuan Xie, Yan Wang, Shangwei Li

**Affiliations:** 1 Division of Reproductive Medical Center, West China Second University Hospital, Sichuan University, Chengdu, Sichuan China; 2 Key Laboratory of Birth Defects and Related Diseases of Women and Children, West China Second University Hospital of Sichuan University, Chengdu, Sichuan China; 3 Department of Cardiology, West China Hospital, Sichuan University, Chengdu, Sichuan China

**Keywords:** Human umbilical cord stem cells, NIV, Frozen-thawed, Human ovarian tissues, In vitro culture

## Abstract

**Background:**

To reduce young female fertility loss, the in-vitro culture of cryopreserved ovarian cortical tissues (OCTs) is considered an effective approach without delaying treatment and undergoing stimulation medicine. However, ischemic damage and follicular loss during the in-vitro culture of OCTs are major technical challenges. Human umbilical cord stem cells (HUMSCs) and their conditioned medium (HUMSC-CM) have been considered to be potential resources for regeneration medicine because they secrete cytokines and enhance cell survival and function. The aim of this study was to determine whether HUMSC-CM improves the development of frozen-thawed in-vitro cultured ovarian tissues compared with a serum-free culture medium (SF-CM).

**Methods:**

The thawed OCTs (*n* = 68) were cultivated in HUMSC-CM and SF-CM in vitro for 8 days, and the ovarian tissues were processed and analyzed by a classical histological evaluation. The microvessel density (MVD) and apotosis detection during in-vitro culture of OCTs were also performed.

**Results:**

A significant difference in the rate of morphologically normal primordial follicles in the HUMSC-CM group was observed compared to that in the SF-CM group (group C) from days 2 to 4 (day 2: group B 58.0 ± 2.45% vs group C 32.0 ± 5.83%, *p* = 0.002; day 3: group B 55.5 ± 4.20% vs group C 21.0 ± 9.80%, *p* = 0.048; day 4: group B 52.0 ± 4.08% vs group C 21.5 ± 8.19%, *p* = 0.019). The microvessel density (MVD) detection showed a time-dependent increase and peaked on day 4. There was a significant difference between groups B (49.33 ± 0.58) and C (24.33 ± 3.79) (*p* = 0.036). The percentage of apoptotic follicles in group B was lower than that in group C on day 1 (13.75 ± 2.50% vs 27.0 ± 10.10%, *p* = 0.003), day 5 (11.75 ± 1.50% vs 51.0 ± 10.5%, *p* = 0.019) and day 7 (15.0 ± 5.10% vs 46.5 ± 21.75%, *p* = 0.018).

**Conclusions:**

These data have provided the first experimental evidence of the effect of HUMSC-CM on frozen-thawed OCTs in vitro. The results showed that the HUMSC-CM group provided a better protecting effect on the in-vitro culture of the cryopreserved OCTs compared to the SF-CM group.

## Background

Aging, gonadotoxic treatments, and certain autoimmune diseases are currently the major reasons for the increased risk of fertility loss [1, 2]. Improvement in reproductive medicine and cryobiology has enabled many females to avoid fertility loss. Compared with embryo and oocyte freezing, ovarian tissue cryopreservation (OTC) could be offered to prepubertal girls, premarital women, or married women who cannot delay cancer treatment or injections of ovarian stimulating medicines [3]. The follicles within the tissues can be initiated to grow towards the formation of fertilizable oocytes with a lesser ethical dilemma. OTC offers the potential to restore natural fertility by thawing and transplanting the ovarian tissues (OTs) or maturing the oocytes in vitro (IVM) to allow their development in the future. Due to the limitation posed by the high risk of malignant recurrence in transplantation, an in-vitro culture is an alternative that appears to be more highly recommended. However, ischemic damage and follicular loss during the in-vitro culture of the cryopreserved OTs remain the main challenges [4].

Nowadays, there is evidence demonstrating that human mesenchymal stem cells (MSCs) are effective in angiogenesis in ischemia animal models and clinical vascular diseases [5, 6]. In particular, human umbilical cord (UC) stem cells (HUMSCs) from umbilical cord Wharton’s jelly are prominent, painless to extract, have high proliferation rates, are more convenient to manipulate with a lower rate of contamination by viruses and bacteria, and satisfy ethical approval [7]. Simultaneously, their features of low immunogenicity, as detected by HLA-DR negative labeling, relative safety, and lack of significant adverse effects have been previously reported [8]. Additionally, the conditioned medium from MSCs has been shown to promote capillary formation in vitro, induce angiogenesis in mice by secreting cytokines and antiapoptotic factors, and enhance cell survival and function [9, 10].

The aim of this study was to determine the effects of a HUMSC conditioned medium (HUMSC-CM) in improving the growth potential of frozen-thawed OCTs in an in-vitro culture compared with those of a serum-free culture medium (SF-CM).

## Methods

### Obtaining, cultivating, and characterizing HUMSCs

UCs were obtained from three healthy donors (aged 27, 28, and 30 years) and washed with phosphate-buffered saline (PBS) to remove the residual blood. Through the removed arteries and veins, the UCs were cut into 1-cm pieces, homogenized to a volume of 1–2 mm^3^, and placed into a HUMSC medium comprising 80% DMEM/F12 (Gibco, USA), 2% fetal bovine serum (FBS; Gibco), 1% insulin-transferrin-selenium (ITS; Sigma, CA), 5 ng/ml epidermal growth factor (EGF; Sigma), and 5 ng/ml basic fibroblast growth factor (b-FGF; Millipore Bioscience, CA) at 37 °C in 5% CO_2_. After the first subculture, the cells were passaged at a ratio of 1:3 every 3 days. HUMSCs before six passages were used in this study.

The second-passage HUMSCs were identified by monoclonal antibodies (Abcam, USA) against CD29, CD31, CD44, CD45, CD105, and HLA-DR incubated at 4 °C for 30 min. Mouse IgG was used as a negative control. The antibody binding was detected by flow cytometry (BD Biosciences, USA). In addition, we performed an alkaline phosphatase (ALP) assay (Abcam), and Alizarin Red S (Millipore) and Oil Red O (Millipore) staining to conduct the osteogenic and adipogenic experiments under an inverted microscope (Nikon Eclipse, TE2000-U).

### Preparation of HUMSC-CM

The early passages of the HUMSCs cells (passages three to five) were cultured in the HUMSC medium until they reached 70–80% confluence in the culture flasks. The medium was then replaced with RPMI 1640 Medium (HyClone, USA), supplemented with 1% antibiotic/antimycotic at 37 °C in 5% CO_2_. After 48 h, the medium was collected, filtered through a 0.22-μM Millex-GP syringe filter (Millipore) and stored at –20 °C until use as the HUMSC-CM.

### Collection of the human ovarian cortex and preparation of human ovarian cortical tissues

Human ovarian cortical tissues (OCTs) were collected from patients who underwent a laparoscopy because of endometriosis or teratoma (mean size 4.26 cm) (Table 1) after informed and approved consent was obtained according to institutional guidelines as reported previously [11]. All OCTs were fixed in 4% formaldehyde (Puzheng Biotech Co., China) for the pathological examination to exclude malignant transformation or metastasis.

**Table 1:** The patients from whom human ovarian tissues were collected

Case	Age (years)	Diagnosis	Treated side	Size (cm)	Date
Sisi Guo	29	Teratoma	Left	4.8 × 3.7 × 4.2	22 August 2016
Defang Pu	30	Endometriosis	Right	6.9 × 3.8 × 6.7	22 August 2016
YI Wang	34	Endometriosis	Right	3.5 × 2.8 × 4.7	18 October 2016
Luxi Feng	37	Endometriosis	Left	3.5 × 4.7 × 3.7	21 November 2016
XiaoPing He	34	Endometriosis	Left	3.5 × 3.7 × 3.7	24 November 2016
*Mean*	32.8	–	–	4.26	–

OCTs were collected from five patients (between 29 and 37 years of age) and transported to the laboratory within 30 min in Leibovitz’s L-15 medium (L-15; Sigma, USA) containing 10% FBS at 2–6 °C. The OCTs were then prepared as 2 × 2 × 1–2-mm fragments after removing the medulla in fresh L-15 at 2–6 °C.

### Needle immersed vitrification procedures

OCTs were cryopreserved by needle immersed vitrification (NIV) following procedures based on our previous studies [12]. Briefly, three to four pieces of ovarian fragments were held in a row by an acupuncture needle (Cloud & Dragon Medical Device Co. Ltd, China) in fresh L-15 at 2–6 °C. The needles were then immersed into an equilibration solution consisting of 7.5% (v/v) ethylene glycol (EG; Sigma) and 7.5% (v/v) dimethyl sulfoxide (DMSO; Sigma) in Dulbecco’s phosphate-buffered saline (DPBS; HyClone, USA) supplement with 20% FBS for 10 min, and then a vitrification solution containing 15% DMSO, 15% EG, and 0.5 M sucrose for 2 min at room temperature. After dehydrating the tissues, the remaining vitrification solution in the needles holding the ovarian fragments was removed by an aseptic absorbent gauze. Finally, the needles were rapidly plunged in liquid nitrogen and maintained in cryovials prefilled with liquid nitrogen. The ovarian fragments were stored for longer than 1 week before use.

### Thawing method

For thawing, the needles holding the ovarian fragments were taken out from the cryovials and were immediately transferred to a 1 M sucrose solution that was prewarmed at 37 °C for 5 min. Then, the fragments were serially transferred to 0.5 and 0.25 M sucrose solutions for 5 min at room temperature, washed three times with L-15 with 20% FBS, and finally incubated for 15–20 min at 37 °C with 5% CO_2_ in an incubator. Four ovarian fragments that were fixed in 4% formaldehyde without any treatment were randomly selected as the free control group (group A).

### In-vitro culture of human ovarian fragments

SF-CM is considered the most suitable system for the in-vitro culture of cryopreserved OCTs and has been used as the primary medium in many studies of in-vitro OT cultures [11, 13–15]. The SF-CM was used as a control treatment in this study and consisted of α-MEM (Gibco) as the basic culture with 10% human serum albumin (HSA; Life Global, USA), 50 μg/ml vitamin C (Sigma), 1% ITS-G (10 μg/ml insulin, 5.5 μg/ml transferrin, and 6.7 ng/ml sodium selenite; Sigma), 0.47 mmol/l pyruvic acid (Sigma), 0.5% antibiotic/antimycotic solution (50 IU/ml penicillin G and 50 μg/ml streptomycin sulfate; HyClone), 2 mmol/l l-glutamine (Sigma), and 0.5 IU/ml recombinant human FSH (Gonal-F; Serono Nordic, USA).

Thawed slices of OCTs (*n* = 68) were transferred into Millicell CM inserts (Millipore) that were precoated with 100 μl GFR Matrigel (BD Bioscience) prediluted 1:3 in α-MEM to support tissue growth and fitted into 24-well plates (Millipore) at 37 °C in 5% CO_2_. The ovarian fragments were randomly divided into the following three groups: (1) free control group (group A, *n* = 4; OTs were fixed in 4% formaldehyde directly); (2) the HUMSC-CM group (group B, *n* = 32; OTs were cultured in the HUMSC-CM (100%) medium); and (3) the SF-CM group (group C, *n* = 32; OTs were cultured in the SF-CM (100%) medium). All ovarian fragments were in-vitro cultured completely for 8 days and four slices were retrieved daily and fixed in 4% formaldehyde for 48 h at 4 °C for classic histology.

### Histological evaluation

#### Follicle counts and morphological analysis

The fixed ovarian fragments were dehydrated, embedded in paraffin, and cut into 5-μm serial sections for routine hematoxylin and eosin (H&E; Beisuo Biotech Company, China) staining. The follicles were categorized and counted in every fifth section in which the oocyte nucleus was observed. Double counting in adjacent sections was avoided. The numbers of follicles at various stages were recorded in a 400-fold field magnification according to the standard protocol [16]. The effects of the HUMSC-CM on the in-vitro culture of the cryopreserved OTs were analyzed histologically using parameters such as the morphology and classification of the follicles, neovascularization, and apoptotic follicles.

#### Immunohistochemistry

OCT angiogenesis and cell apoptosis were evaluated by CD34 (rabbit monoclonal, 1:100 dilution; Abcam, UK) staining of epithelial cells in microvessels and active caspase-3 (AC-3; rabbit monoclonal, 1:100 dilution; CST, USA) staining in apoptotic follicles following the manufacturer’s instructions. Human gastric carcinomas and etoposide-treated Jurkat cells were used as positive controls for the CD34 and AC-3 immunohistochemistry, respectively. The primary antibody was omitted from the negative control. The microvessel density (MVD) was calculated as the mean of the CD34 positively stained cells at three randomly selected positions in a high-power field (×400) of each fragment under a light microscope (Olympus BX-51). Meanwhile, the number of apoptotic follicles that were stained in dark brown coloring in the cytoplasm/nucleus of stromal cells, granulosa cells, or oocytes were recorded in the same high-power field (×400), which was randomly selected. The results of the immunohistochemistry were evaluated by XHS and XCX, who were blinded to the conditions.

### Statistical analysis

The experiments were repeated three times unless otherwise specified. The differences between the groups were analyzed by an independent sample *t* test using the SPSS Statistics 17.0 program software (SPSS, Inc., Chicago, IL, USA). The data are described as the mean value ± standard deviation (SD) in all groups. *p* < 0.05 was considered statistically significant.

## Results

### Obtaining, cultivating, and characterizing HUMSCs

**Fig. 1:**
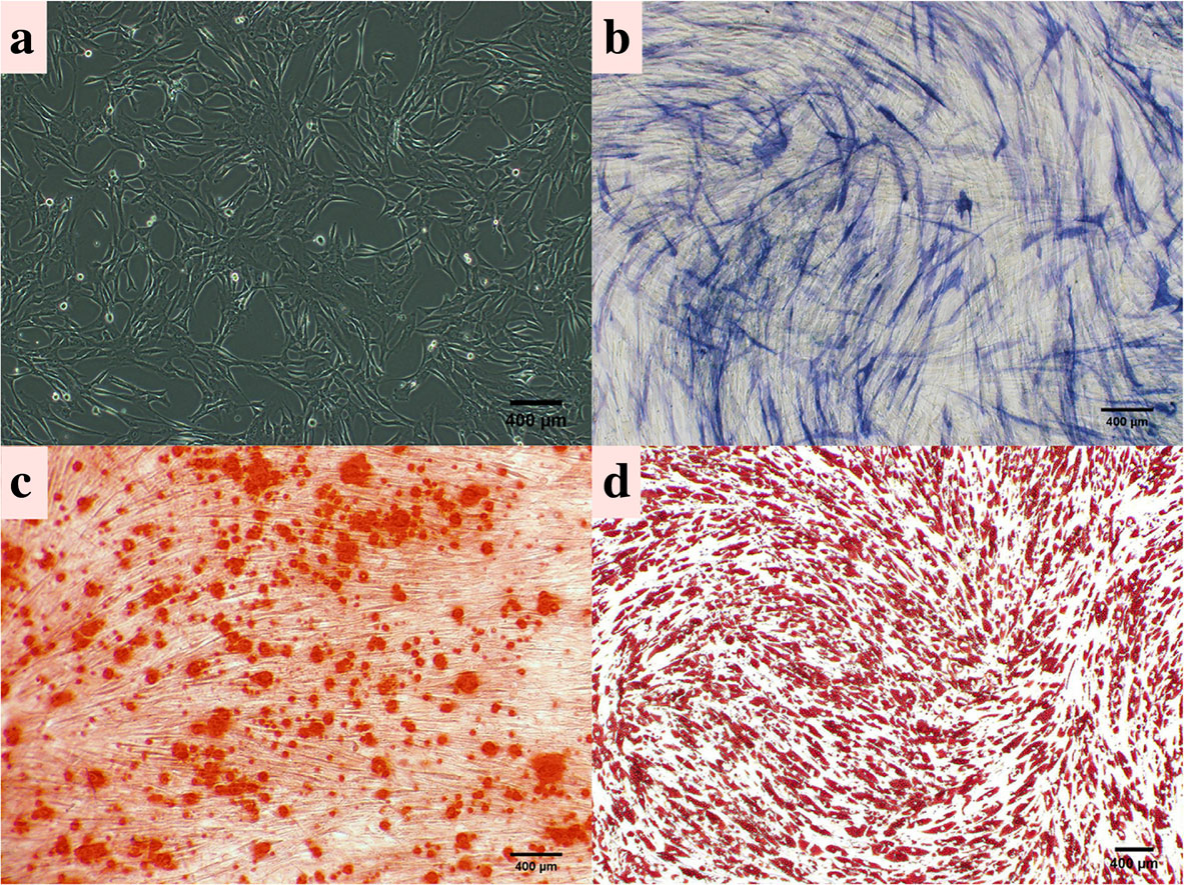
Isolation and cultivation of HUMSCs. **a** Representative image of HUMSC third passages (day 2) (original magnification × 100, *scale bar* = 400 μm). **b** Representative image of the differentiation of the osteoblasts (ALP activity staining) on day 4 after the osteogenic induction. **c** Representative image of the mineralized cell nodules (Alizarin Red S staining) 3 weeks after the osteogenic induction. **d** Representative image of lipid droplets (stained with Oil Red O) 3 weeks after the adipogenic induction (original magnification × 100, *scale bar* = 400 μm)

**Fig. 2:**
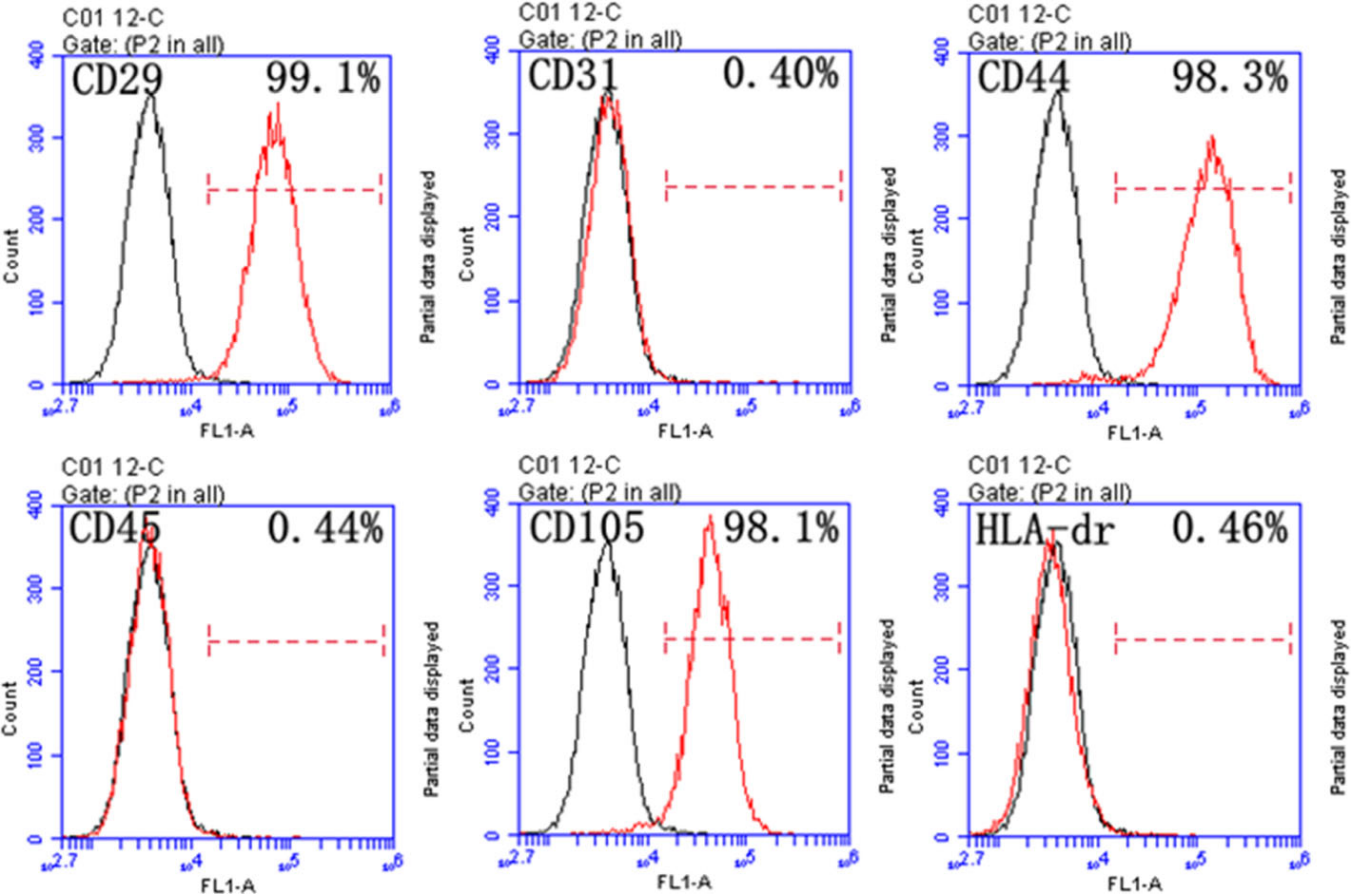
Identification of HUMSCs. Second-passage HUMSCs were identified using monoclonal antibodies. Cell surface markers were identified through flow cytometric analysis

Primary cells were successfully isolated from the umbilical cords and, after 72 h of adherent growth, the cells morphologically resembled fibroblasts and became confluent (Fig. 1a). Meanwhile, the isolated cells were characterized by the surface antigens in the HUMSCs by flow cytometry, such as CD29^+^, CD31^–^, CD44^+^, CD45^–^, CD105^+^, and HLA-DR^–^ (Fig. 2). Furthermore, as an initial indicator of osteoblast differentiation, the ALP activity of the cells was determined 4 days after the blue staining. In addition, after culturing in osteogenic and adipogenic differentiation media for 21 days, the osteocytes that embraced the mineral deposits and appeared orange-red were investigated by an Alizarin Red S assay, and the adipocytes and lipid droplets of the adipocytes that appeared red were detected by an Oil Red O assay (Fig. 1b, c and d).

### Histological evaluation of the OCTs

#### Morphology and classification of the follicles

**Fig. 3:**
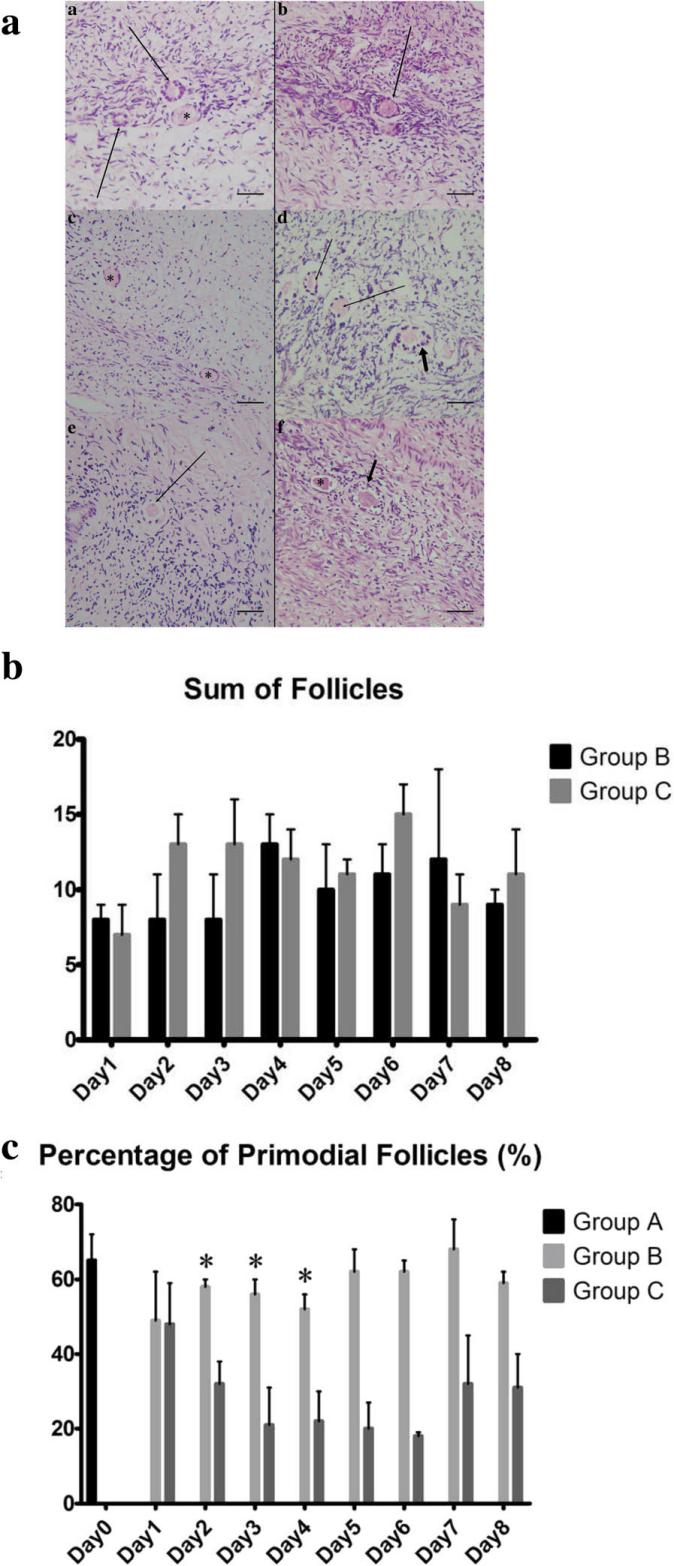
Histological analysis of the thawed OTs. **a** The morphologically normal primordial follicles (*), primary follicles (*thin arrow*), and secondary follicles (*thick arrow*) stained with H&E in the thawed OTs in groups A (a, b), B (c, d) and C (e, f) (original magnification × 400, *scale bar* = 100 μm). **b** Sum of PF as evaluated by H&E staining over 8 days between groups B and C. **c** Percentage of PF as evaluated by H&E staining before and after the in-vitro culture at nine time points in the three groups. *p < 0.05, versus group C

Through the NIV method and thawing, a number of follicles in different developmental stages were observed in the tissues by H&E staining at a high magnification (×400), and representative images are shown in Fig. 3a. In the free control group (group A), no secondary follicles or antral follicles were observed. Similarly, pre-antral follicles were preserved in good morphology after the cryopreservation in the HUMSC-CM group (group B) and SF-CM group (group C). There were no significant differences between groups B and C in the sum of follicles at each time-point (*p* > 0.05) (Fig. 3b). In contrast, there was a significant difference in percentage of primordial follicles (PF) in group B compared to that in group C on 3 of the earlier days (days 2 to 4) (day 2: group B 58.0 ± 2.45% vs group C 32.0 ± 5.83%, *p* = 0.002; day 3: group B 55.5 ± 4.20% vs group C 21.0 ± 9.80%, *p* = 0.048; day 4: group B 52.0 ± 4.08% vs group C 21.5 ± 8.19%, *p* = 0.019). After day 4, no difference could be found between groups B and C (Fig. 3c).

#### HUMSC-CM promoted OT neoangiogenesis

**Fig. 4:**
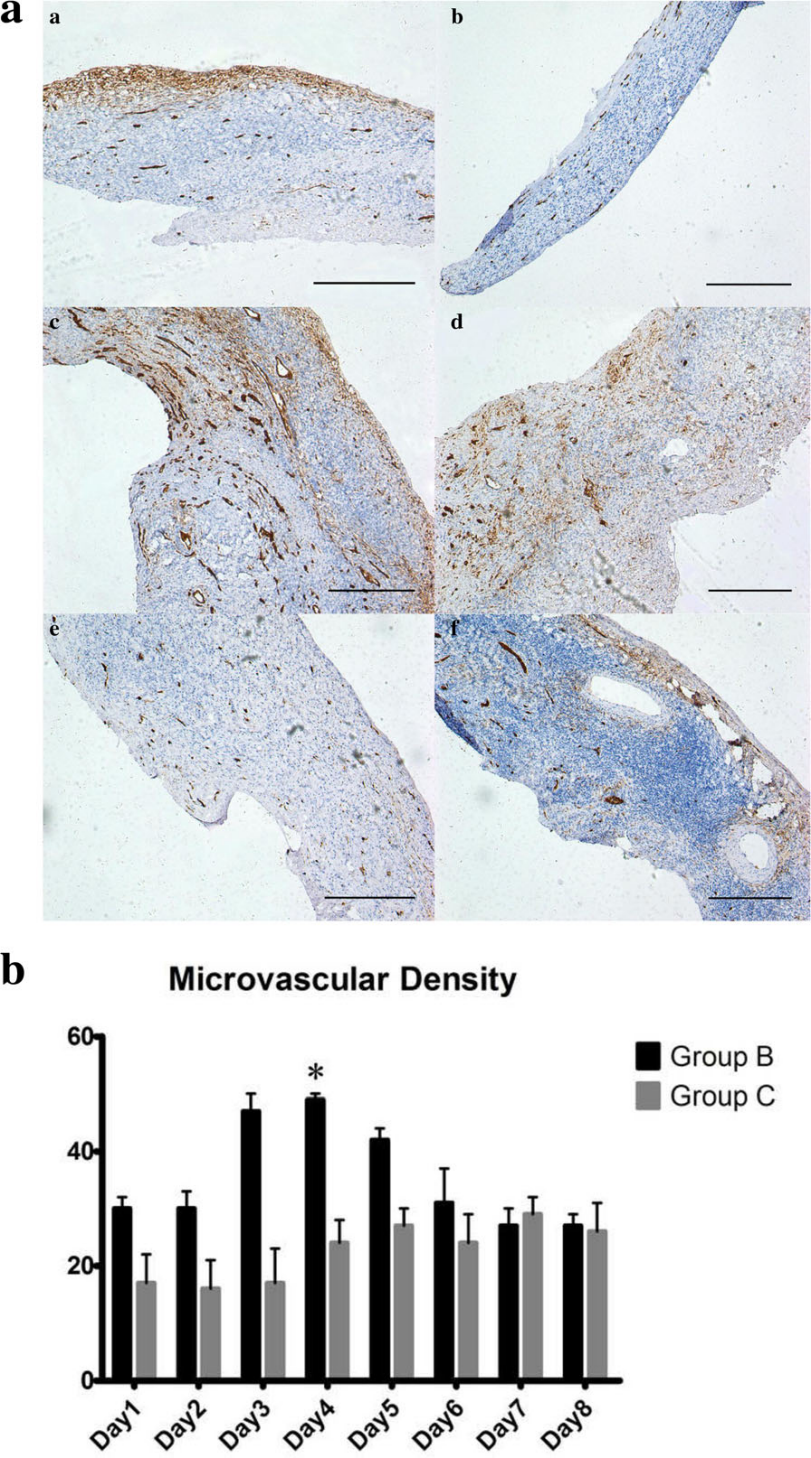
HUMSC-CM promoted neoangiogenesis as detected by CD34 immunohistochemistry over 8 days. **a** Representative images of CD34 immunohistochemistry staining of OTs in groups B (*left*) (a, c, and e) and C (*right*) (b, d, and f) on days 1, 4, and 8 (original magnification × 100, *scale bar* = 400 μm). **b** MVD evaluated by CD34-positive staining over 8 consecutive days is shown in the two groups. *p < 0.05, versus group C

The CD34 marker is reliably detected in the human ovary as a transmembrane glycoprotein that is localized in endothelial cells. In addition, MVD was used to examine the neoangiogenesis activity of the tissues. As shown in Fig. 4a, there was a general time-dependent increase in the microvascular formation in the first 5 days. Then, the MVD decreased and maintained a stable level in both groups (groups B and C). On day 4, the MVD in group B (49.33 ± 0.58) reached a peak and was apparently higher than that in group C (24.33 ± 3.79; *p* = 0.036) (Fig. 4b), although the data were not comparable on the other days (Fig. 4b).

#### HUMSC-CM inhibited follicular apoptosis

**Fig. 5:**
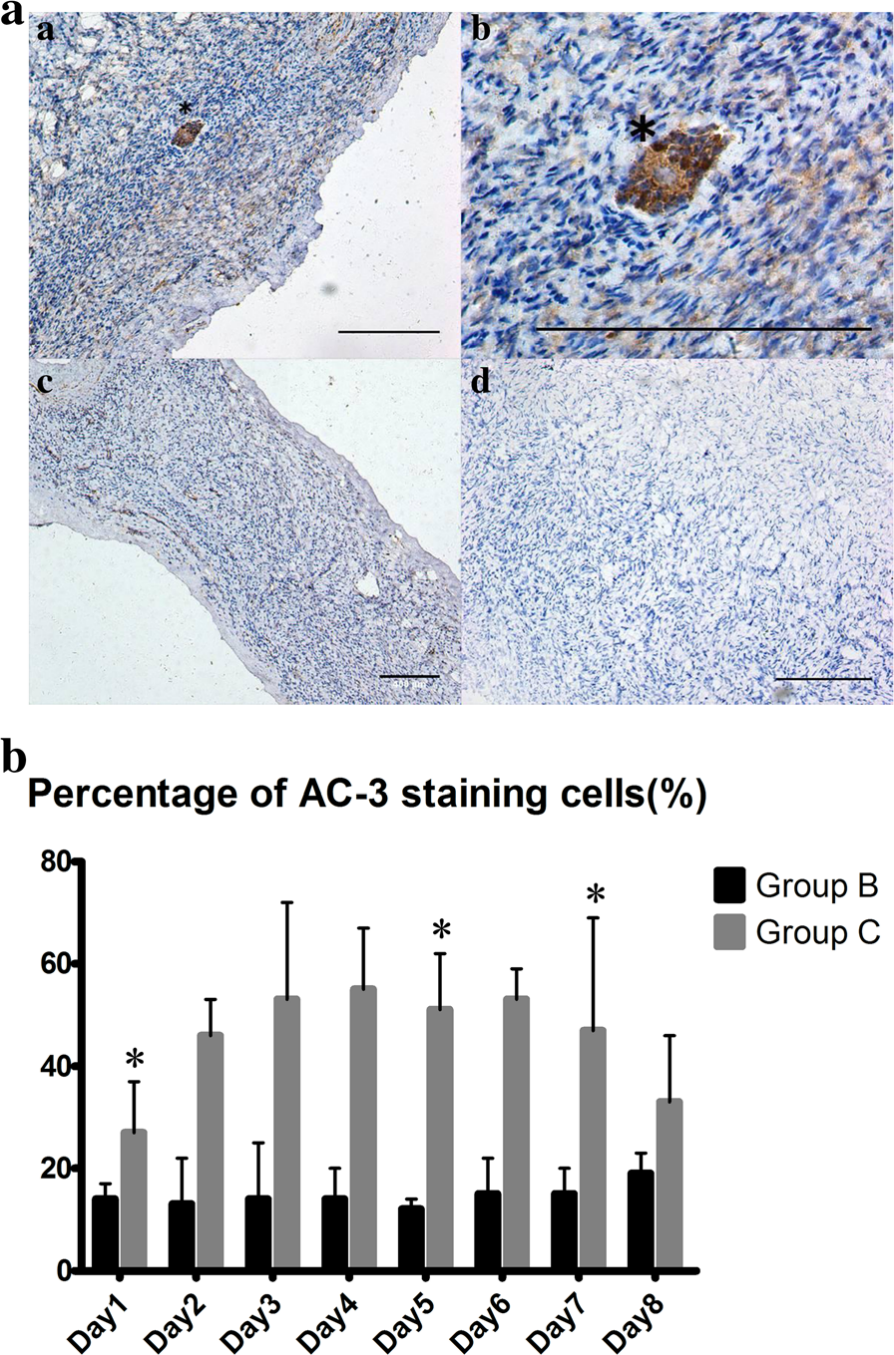
HUMSC-CM inhibited follicular apoptosis as detected by active caspase-3 (*AC-3*) immunohistochemistry over 8 days. **a** Representative images of the AC-3 immunohistochemistry staining of the follicles. a and b: Apoptosis was observed in the morphologically normal PF (*); c and d: Follicular apoptosis was not observed (×100 (c), ×200 (a, d), ×400 (d), *scale bar* = 400 μm). **b** Percentage of AC-3 positively stained follicles over 8 consecutive days is shown in the two groups. *p < 0.05, versus group B

Follicular apoptosis was shown by AC-3-positive staining, resulting in follicles that were dark brown in color, and most of the follicles were PF as shown by the representative images in Fig. 5a. Compared with group C, the percentage of apoptotic follicles on 3 of the days (days 1, 5, and 7) appeared to be dramatically decreased in group B (day 1: group B 13.75 ± 2.50% vs group C 27.0 ± 10.10%, *p* = 0.003; day 5: group B 11.75 ± 1.50% vs group C 51.0 ± 10.5%, *p* = 0.019; day 7: group B 15.0 ± 5.10% vs group C 46.5 ± 21.75%, *p* = 0.018) (Fig. 5b).

## Discussion

Human OTs containing immature PF have been successfully cryopreserved to preserve fertility with a lesser ethical dilemma for women facing a reproductive compromise. OCT has allowed for the development of alternative strategies for the re-establishment of fertility in women at a risk of fertility loss, particularly those that underwent cancer treatment [17, 18]. Although autotransplantation, which is an OCT approach, is limited by the high risk of a malignant re-transplantation, an in-vitro culture is an alternative approach that appears to be more highly recommended. Promoting angiogenesis and neovascularization were not only the most important achievements in the in-vitro culture [4], but were also the primary difficulties in OCT.

Many tissue repair studies have explored the paracrine activity and proliferation of HUMSCs [19, 20]. These studies suggested that MSCs exert their protective effects via paracrine-distinct growth factors and cytokines, such as vascular endothelial growth factor (VEGF), hepatocyte growth factor (HGF), and insulin-like growth factor (IGF)-1, against several diseases or to repair tissue damage [21]. There were also several studies in the reproductive area that demonstrated that HUMSC transplantation improves ovarian function and prohibits follicular apoptosis due to ovarian damage caused by superovulation or chemotherapy [16, 22]. The positive factors secreted by the transplanted HUMSCs, such as VEGF, IGF-1, and HGF, acted as antiapoptosis-related factors, although no studies have found HUMSCs to develop into follicular components [6]. These results indicated that the antiapoptotic effect of the transplantation of HUMSCs primarily manifests in the protection of the secondary follicles and antral follicles [16]. The current results showed that the AC-3-positive follicles were mostly in the PF. The conditioned medium of MSCs was also found to promote capillary formation, induce angiogenesis, and exert a therapeutic function. Lotfinia et al. indicated that the HUMSC-CM contained a higher concentration of VEGF, and the results suggested that HUMSC-CM treatment may enhance vascular formation in ischemic rats [23]. However, it is unclear whether HUMSC-CM can improve the development of the cryopreserved OCTs in an in-vitro culture system. Thus, by comparing HUMSC-CM to SF-CM in OT in-vitro culture, the SF-CM was suggested as an organ culture system for human follicles yielding a reduced apoptotic rate and better follicular survival, particularly in tissues that are cut into cubes [24]. In this study, a 1640 medium was applied to prepare the serum-free HUMSC-CM, which efficiently avoided the effect of FBS.

NIV is a new vitrification technique that can maintain the satisfactory viability of the ovarian follicles and stroma compared to another cryopreservation methods that was used in a previous study [12, 25]. In this study, NIV was capable of reproducible preservation as evaluated by H&E staining which showed a positive structure of the follicles and a better percentage of PF in group A (64.75 ± 7.23%) (Fig. 3b). Notably, there were no secondary follicles or antral follicles observed in group A, while no antral follicles were observed in groups B and C. We speculated that the cause was the sensitivity of antral follicles to cryoinjury. During the cryopreservation of the OTs, cryoinjury inevitably occurred which had a detrimental effect on the ovarian quality and reserve [26]. Thus, restoring the function and structure of the stroma of thawed OTs in an in-vitro culture system was important. In addition, developing follicles requires a lengthy and complicated process in humans, and it is difficult to simulate the natural system in vitro. Overcoming the limit of follicular growth and death in current in-vitro systems requires a better proportion of the activation of follicle growth initiation, the interplay of important growth factors, the basic metabolic needs of early growing follicles, and mimicking the natural environment of the follicles. Furthermore, an ischemic injury that lacks a supportive and sustaining bloodstream limits the development of PFs only to the pre-antral stage [27]. However, in a DEHP-induced OT study, the extra accumulation of reactive oxygen species (ROS) produced by the oxidative stress system was found to lead to follicular apoptosis and affect angiogenesis [28]. Thus, this result suggests that apoptosis occurred throughout the entire study, and we consider that follicular loss and stroma integrity damage can be the result of a lack of a sufficient blood supply in vitro [4, 11]. Therefore, we investigated ovarian follicle counts at various stages, MVD, and percentage of apoptosis.

Based on our results, in groups B and C there was a time-dependent generation of neoangiogenesis that gradually increased until day 5, after which it declined to a stable level. In particular, the neoangiogenic capability of the HUMSC-CM peaked on day 4. The neoangiogenic effect on days 3 to 5 was remarkable in both groups, and another OT transplantation study reported that the initial revascularization of human OT occurred within 3 to 5 days [29]. This result indicated that neoangiogenesis of human thawed OTs in vitro occurred earlier than that in transportation. In humans, a period of hypoxia occurred in the thawed OTs that was found to have an effect on oxygenation during the active process of graft revascularization [30]. This agrees with our MVD results over the first 5 days. However, as stated above, ROS from the oxidative stress system affected angiogenesis, which might lead a decline in MVD after day 5 in this study. Based on these results, we might presume an oxidative stress-induced process of revascularization on OTs in vitro. When outside of their natural environment, follicles must cope with physical restrictions, such as oxygen tension, which likely stimulates neoangiogenesis more rapidly than in transplantation.

In addition, over 8 consecutive days, the percentage of PF corresponded to the neoangiogenesis detected by MVD. This result suggests that promoting the microvascular formation led to an improved follicular development. Interestingly, there was a significant difference in the developed PF and MVD between groups B and group C on day 4, followed by a significant difference in apoptotic reduction on day 5. This result suggests that promoting microvascular formation occurred earlier than the prohibition of apoptotic follicles in the in-vitro system. We speculate that the HUMSC-CM contains angiogenic factors that are secreted by the HUMSCs, such as b-FGF and VEGF, and that change the vascular microenvironment to function as “drugstores” surrounding the follicles, which results in a reduction in the apoptosis of ovarian follicles [31]. This result is consistent with a previous study [31] where applied MSCs in human follicle isolation in-vitro culture decreased follicular apoptosis and promoted follicular development. Compared to SF-CM, the number of AC-3 positively stained cells decreased significantly after the HUMSC-CM treatment. Based on these results, there is a positive role of HUMSC-CM in the thawed OTs in vitro. Unfortunately, our study was a prospective research study that investigated the effect of the HUMSC-CM on thawed OTs in an in-vitro system. Thus, future studies on HUMSC-CM should be conducted to better understand the beneficial and protective effects of the CM during OCT in-vitro culture progress.

The results of these experiments indicate that HUMSC-CM played a cytoprotective role and had high antiapoptotic properties compared to SF-CM. The HUMSC-CM components that participated in the protective process might be associated with the paracrine activity of HUMSCs.

## Conclusions

Overall, we firstly used HUMSC-CM to improve the recovery of frozen-thawed OCTs in the in-vitro culture system The results revealed features promoting microvascular formation and inhibiting follicular apoptosis by the HUMSC-CM treatment and demonstrated the potential application of HUMSCs and their conditioned medium in the area of reproductive medicine in the future.

## Abbreviations

AC-3: Active caspase-3
ALP: Alkaline phosphatase
b-FGF: Basic fibroblast growth factor
DMSO: Dimethyl sulfoxide
DPBS: Dulbecco phosphate-buffered saline
EG: Ethylene glycol
EGF: Epidermal growth factor
FBS: Fetal bovine serum
H&E: Hematoxylin and eosin
HGF: Hepatocyte growth factor
HSA: Human serum albumin
HUMSC: Human umbilical cord stem cell
HUMSC-CM: Human umbilical cord stem cell conditioned medium
IGF: Insulin-like growth factor
ITS: Insulin-transferrin-selenium
IVM: In vitro maturation
L-15: Leibovitz’s L-15 medium
MSC: Mesenchymal stem cell
MVD: Microvessel density
NIV: Needle immersed vitrification
OCT: Ovarian cortical tissue
OT: Ovarian tissue
OTC: Ovarian tissue cryopreservation
PF: Primordial follicles
ROS: Reactive oxygen species
SF-CM: Serum-free culture medium
UC: Umbilical cord
VEGF: Vascular endothelial growth factor
